# Reversal of cognitive decline: A novel therapeutic program

**DOI:** 10.18632/aging.100690

**Published:** 2014-09-27

**Authors:** Dale E. Bredesen

**Affiliations:** ^1^ Mary S. Easton Center for Alzheimer's Disease Research, Department of Neurology, University of California, Los Angeles, CA 90095;; ^2^ Buck Institute for Research on Aging, Novato, CA 94945

**Keywords:** Alzheimer's, dementia, mild cognitive impairment, neurobehavioral disorders, neuroinflammation, neurodegeneration, systems biology

## Abstract

This report describes a novel, comprehensive, and personalized therapeutic program that is based on the underlying pathogenesis of Alzheimer's disease, and which involves multiple modalities designed to achieve metabolic enhancement for neurodegeneration (MEND). The first 10 patients who have utilized this program include patients with memory loss associated with Alzheimer's disease (AD), amnestic mild cognitive impairment (aMCI), or subjective cognitive impairment (SCI). Nine of the 10 displayed subjective or objective improvement in cognition beginning within 3-6 months, with the one failure being a patient with very late stage AD. Six of the patients had had to discontinue working or were struggling with their jobs at the time of presentation, and all were able to return to work or continue working with improved performance. Improvements have been sustained, and at this time the longest patient follow-up is two and one-half years from initial treatment, with sustained and marked improvement. These results suggest that a larger, more extensive trial of this therapeutic program is warranted. The results also suggest that, at least early in the course, cognitive decline may be driven in large part by metabolic processes. Furthermore, given the failure of monotherapeutics in AD to date, the results raise the possibility that such a therapeutic system may be useful as a platform on which drugs that would fail as monotherapeutics may succeed as key components of a therapeutic system.

## INTRODUCTION

### 

#### Magnitude of the problem

Cognitive decline is a major concern of the aging population, and Alzheimer's disease is the major cause of age-related cognitive decline, with approximately 5.4 million American patients and 30 million affected globally [1]. In the absence of effective prevention and treatment, the prospects for the future are of great concern, with 13 million Americans and 160 million globally projected for 2050, leading to potential bankruptcy of the Medicare system. Unlike several other chronic illnesses, Alzheimer's disease prevalence is on the rise, which makes the need to develop effective prevention and treatment increasingly pressing. Recent estimates suggest that AD has become the third leading cause of death in the United States [2], behind cardiovascular disease and cancer. Furthermore, it has been pointed out recently that women are at the epicenter of the Alzheimer's epidemic, with 65% of patients and 60% of caregivers being women [3]. Indeed, a woman's chance of developing AD is now greater than her chance of developing breast cancer [4].

#### Failure of monotherapeutics

Neurodegenerative disease therapeutics has been, arguably, the field of greatest failure of biomedical therapeutics development. Patients with acute illnesses such as infectious diseases, or with other chronic illnesses, such as cardiovascular disease, osteoporosis, human immunodeficiency virus infection, and even cancer, have access to more effective therapeutic options than do patients with AD or other neurodegenerative diseases such as Lewy body dementia, frontotemporal lobar degeneration, and amyotrophic lateral sclerosis. In the case of Alzheimer's disease, there is not a single therapeutic that exerts anything beyond a marginal, unsustained symptomatic effect, with little or no effect on disease progression. Furthermore, in the past decade alone, hundreds of clinical trials have been conducted for AD, at an aggregate cost of billions of dollars, without success. This has led some to question whether the approach taken to drug development for AD is an optimal one.

Therapeutic success for other chronic illnesses such as cardiovascular disease, cancer, and HIV, has been improved through the use of combination therapies [5]. In the case of AD and its predecessors, mild cognitive impairment (MCI) and subjective cognitive impairment (SCI), comprehensive combination therapies have not been explored. However, the past few decades of genetic and biochemical research have revealed an extensive network of molecular interactions involved in AD pathogenesis, suggesting that a network-based therapeutics approach, rather than a single target-based approach, may be feasible and potentially more effective for the treatment of cognitive decline due to Alzheimer's disease.

#### Preclinical studies

Extensive preclinical studies from numerous laboratories have identified multiple pathogenetic targets for potential intervention. These include, in addition to amyloid-β (Aβ) oligomers and tau, inflammatory mediators, apolipoproteins and lipid metabolism factors, hormonal mediators, trophic factors and their receptors, calcium regulatory pathways, axoplasmic transport machinery, neurotransmitters and their receptors, prion protein, and a host of other potential targets. However, one of the drawbacks of these preclinical studies is that many have implicated single pathways, and shown large effects of targeting one pathway, whereas in human studies, such approaches have not been borne out. There are several possible inferences from such discrepant results: first, it is possible that it will be necessary to target multiple pathways simultaneously in order to effect an improvement in symptoms and pathophysiology. Second, it is possible that targeting a single pathway will be sufficient, but that earlier intervention will be required. Third, it is possible that all of these seemingly disparate pathways will converge on a single critical pathway, so that either a single targeted therapy or a multi-component, multi-targeted approach may be effective. And fourth, of course it is possible that neither of these two types of approaches will be sufficient. It is worth noting, however, that it is possible that addressing multiple targets within the network underlying AD pathophysiology may be successful even when each target is affected in a relatively modest way; in other words, the effects of the various targets may be additive, multiplicative, or otherwise synergistic.

**Figure 1:**
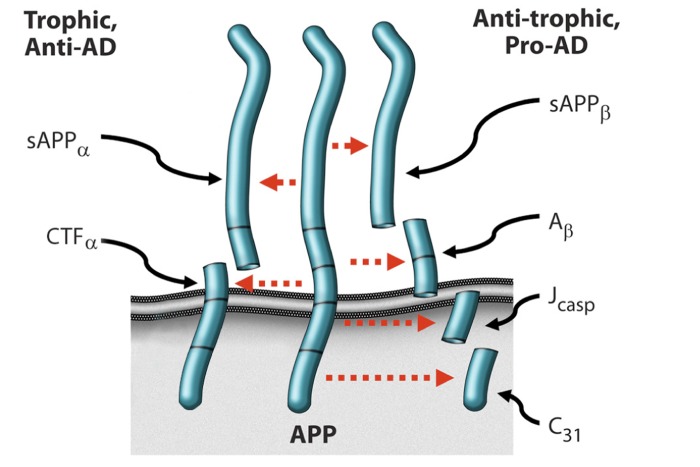
Alternative processing of, and signaling by, APP. [5].

Based on a combination of in vitro and in vivo studies, we have advanced a model in which AD results from an imbalance in endogenous plasticity signaling (Fig. 1), 5-9, and in which the β-amyloid precursor protein (APP) is a mediator of such plasticity-related signaling. Thus the model suggests that AD is analogous to other chronic illnesses such as cancer, osteoporosis, and atherosclerosis. In the case of osteoporosis, osteoblastic signaling is chronically exceeded by osteoclastic signaling, resulting in an age-associated chronic illness featuring loss of bone. By analogy, in Alzheimer's disease, there is a fundamental, age-associated imbalance between the dynamically opposed physiological processes that mediate plasticity, i.e., between synaptoblastic and synaptoclastic activity. This signaling involves physiological mediators of synaptic development, maintenance, repair, and remodeling, including APP, its derivative peptides, ApoE, and tau, and is modulated by all of the many disparate factors associated with Alzheimer's disease. Furthermore, just as for neoplasia, positive feedback selects and amplifies the disease process; however, whereas in oncogenesis, the positive feedback occurs at the cellular level, in Alzheimer's disease, the positive feedback occurs at the molecular species level, in the form of prionic loops [5, 8, 9].

In support of this model, the four peptides derived from the amyloidogenic processing of β-amyloid precursor protein (APP)—sAPPβ, Aβ, Jcasp, and C31—have been shown to mediate neurite retraction, synaptic inhibition, caspase activation, and programmed cell death [6, 10-12]; whereas, in contrast, the two peptides derived from the non-amyloidogenic processing of APP—sAPPα and αCTF—mediate neurite extension, and inhibit Aβ production, caspase activation, and programmed cell death 13-15. Thus APP appears to function as a molecular switch, mediating plasticity-related processes, and AD is associated, whether causally or incidentally, with an increase in the ratio of the neurite-retractive peptides to the neurite-extending peptides. Reducing this ratio, whether by affecting BACE (β-site APP cleaving enzyme) or other cleavage of APP, appears to mitigate the AD severity [7, 16, 17].

Of particular interest for the development of a therapeutic program whose goal is to correct the hypothesized chronic synaptoblastic:synaptoclastic imbalance is the feedback mechanism: whereas homeostatic (negative) feedback is utilized by biological systems with single goal outcomes (e.g., serum pH) and no requirement for amplification, prionic loop (positive) feedback is utilized by biological systems with multi-goal outcomes and a requirement for rapid amplification (e.g., thrombus formation or, potentially, synapse modulation), and such systems therefore function as molecular switches [9]. In these latter systems, the positive feedback feature of the systems dictates that the molecular mediators involved, or a subset thereof, beget more of themselves, or enhance their own activities. Thus such amplifying systems are prionic, with the degree of infectivity depending on the stability of the molecular species involved. In the case of APP signaling, binding of a trophic ligand such as netrin-1 increases the production of sAPPα [18], which inhibits BACE cleavage [19], with the complementary fragment, αCTF, inhibiting γ-secretase cleavage [14]; thus cleavage at the α-site produces fragments that inhibit cleavage at the β-site and γ-site rather than feeding back to reduce α-site cleavage. Similarly, cleavage at the β-site and γ-site to produce Aβ feeds back positively to increase APP-C31 production [20], thus favoring the pro-AD, anti-trophic processing of APP. Moreover, Aβ itself has been shown to exhibit prionic properties [21], although the mechanism by which it does so has not been clarified.

Thus APP processing displays positive feedback, and therefore APP and its derivative peptides function as a molecular switch. This has critical implications for therapeutic development, since it offers a mechanism by which a threshold effect occurs. We have taken advantage of this phenomenon to develop drug candidates that increase the anti-AD, trophic APP signaling, while reducing the pro-AD, anti-trophic APP signaling [22] and enhancing cognition [23].

We have found that the manipulation of the plasticity balance that is mediated or reflected by the APP-derivative peptide balance (Fig. 1), whether genetically or pharmacologically, leads to predictable effects on learning and memory. Mutation of the caspase site at Asp664 inhibits the synaptic loss, memory deficits, and dentate gyral atrophy that otherwise occurs in the PDAPP transgenic mouse model of AD [7, 17, 24-26]. Furthermore, knock-in studies of a wild type mouse D664A support the notion that APP is indeed involved fundamentally in plasticity. (Kane, et al, unpublished data, 2014)

#### Systems biology and systems therapeutics of AD

The transgenic mouse studies suggest that APP signaling can be manipulated to inhibit AD pathophysiology. However, the mouse models feature mutations in APP or other familial AD-related genes such as presenilin-1, whereas the large majority of patients with AD suffer from sporadic AD, without an APP or PS1 mutation (although the majority do express the ε4 allele of ApoE). Given the many inputs to the APP signaling balance in humans (e.g., estrogen, netrin-1, Aβ, etc.), and the minimal success with each of many potentially therapeutic agents (e.g., estrogen, melatonin, exercise, vitamin D, curcumin, *Ashwagandha*, etc.), the pathobiology of AD dictates a system or program rather than a single targeted agent. Successes with other chronic illnesses such as cardiovascular disease, neoplasia, and HIV support the efficacy of multiple-component systems. My colleague and I have recently described such a system for AD [5]. The basic tenets for such a comprehensive therapeutic system are the following:

1. Just as for other chronic illnesses such as atherosclerotic cardiovascular disease, the goal is not simply to normalize metabolic parameters, but rather to optimize them. As an example, a serum homocysteine level of 12 μmol/l is considered to be within normal limits, but is well documented to be suboptimal [27]. Similar arguments can be made for many other metabolic parameters.
2. Based on the hypothesis that AD results from an imbalance in an extensive plasticity network, the therapy should address as many of the network components as possible, with the idea that a combination may create an effect that is more than the sum of the effects of many monotherapeutics [5].
3. Just as for other chronic illnesses such as osteoporosis, cancer, and cardiovascular disease, the underlying network features a threshold effect, such that, once enough of the network components have been impacted, the pathogenetic process would be halted or reversed. Therefore, even though it is not expected that most patients will be able to follow every single step of the protocol, as long as enough steps are followed to exceed the threshold, that should be sufficient.
4. The approach is personalized, based on the contributory laboratory values affecting the plasticity network; and is computationally intensive, since many physiological data points are analyzed, interdependent network-component status is assessed, and many interventions are prioritized to determine the therapeutic program.
5. The program is iterative, so that there is continued optimization over time.
6. For each network component, the goal is to address it in as physiological a way, and as far upstream, as possible.

## RESULTS

### CASE STUDIES

#### Patient one: history

A 67-year-old woman presented with two years of progressive memory loss. She held a demanding job that involved preparing analytical reports and traveling widely, but found herself no longer able to analyze data or prepare the reports, and therefore was forced to consider quitting her job. She noted that when she would read, by the time she reached the bottom of a page she would have to start at the top once again, since she was unable to remember the material she had just read. She was no longer able to remember numbers, and had to write down even 4-digit numbers to remember them. She also began to have trouble navigating on the road: even on familiar roads, she would become lost trying to figure out where to enter or exit the road. She also noticed that she would mix up the names of her pets, and forget where the light switches were in her home of years.

Her mother had developed similar progressive cognitive decline beginning in her early 60s, had become severely demented, entered a nursing home, and died at approximately 80 years of age. When the patient consulted her physician about her problems, she was told that she had the same problem her mother had had, and that there was nothing he could do about it. He wrote “memory problems” in her chart, and therefore the patient was turned down in her application for long-term care.

After being informed that she had the same problem as her mother had had, she recalled the many years of her mother's decline in a nursing home. Knowing that there was still no effective treatment and subsequently losing the ability to purchase long-term care, she decided to commit suicide. She called a friend to commiserate, who suggested that she get on a plane and visit, and then referred her for evaluation.

She began System 1.0 (Table 1), and was able to adhere to some but not all of the protocol components. Nonetheless, after three months she noted that all of her symptoms had abated: she was able to navigate without problems, remember telephone numbers without difficulty, prepare reports and do all of her work without difficulty, read and retain information, and, overall, she became asymptomatic. She noted that her memory was now better than it had been in many years. On one occasion, she developed an acute viral illness, discontinued the program, and noticed a decline, which reversed when she reinstated the program. Two and one-half years later, now age 70, she remains asymptomatic and continues to work full-time.

**Table 1:** Therapeutic System 1.0

Goal	Approach	Rationale and References
Optimize diet: minimize simple CHO, minimize inflammation.	Patients given choice of several low glycemic, low inflammatory, low grain diets.	Minimize inflammation, minimize insulin resistance.
Enhance autophagy, ketogenesis	Fast 12 hr each night, including 3 hr prior to bedtime.	Reduce insulin levels, reduce Aβ.
Reduce stress	Personalized—yoga or meditation or music, etc.	Reduction of cortisol, CRF, stress axis.
Optimize sleep	8 hr sleep per night; melatonin 0.5mg po qhs; Trp 500mg po 3x/wk if awakening. Exclude sleep apnea.	[36]
Exercise	30-60′ per day, 4-6 days/wk	[37, 38]
Brain stimulation	Posit or related	[39]
Homocysteine <7	Me-B12, MTHF, P5P; TMG if necessary	[40]
Serum B12 >500	Me-B12	[41]
CRP <1.0; A/G >1.5	Anti-inflammatory diet; curcumin; DHA/EPA; optimize hygiene	Critical role of inflammation in AD
Fasting insulin <7; HgbA1c <5.5	Diet as above	Type II diabetes-AD relationship
Hormone balance	Optimize fT3, fT4, E2, T, progesterone, pregnenolone, cortisol	[5, 42]
GI health	Repair if needed; prebiotics and probiotics	Avoid inflammation, autoimmunity
Reduction of A-beta	Curcumin, Ashwagandha	43-45
Cognitive enhancement	Bacopa monniera, MgT	[46, 47]
25OH-D3 = 50-100ng/ml	Vitamins D3, K2	[48]
Increase NGF	H. erinaceus or ALCAR	[49, 50]
Provide synaptic structural components	Citicoline, DHA	[51].
Optimize antioxidants	Mixed tocopherols and tocotrienols, Se, blueberries, NAC, ascorbate, α-lipoic acid	[52]
Optimize Zn:fCu ratio	Depends on values obtained	[53]
Ensure nocturnal oxygenation	Exclude or treat sleep apnea	[54]
Optimize mitochondrial function	CoQ or ubiquinol, α-lipoic acid, PQQ, NAC, ALCAR, Se, Zn, resveratrol, ascorbate, thiamine	[55]
Increase focus	Pantothenic acid	Acetylcholine synthesis requirement
Increase SirT1 function	Resveratrol	[32]
Exclude heavy metal toxicity	Evaluate Hg, Pb, Cd; chelate if indicated	CNS effects of heavy metals
MCT effects	Coconut oil or Axona	[56]

CHO, carbohydrates; Hg, mercury; Pb, lead; Cd, cadmium; MCT, medium chain triglycerides; PQQ, polyquinoline quinone; NAC, N-acetyl cysteine; CoQ, coenzyme Q; ALCAR, acetyl-L-carnitine; DHA, docosahexaenoic acid; MgT, magnesium threonate; fT3, free triiodothyronine; fT4, free thyroxine; E2, estradiol; T, testosterone; Me-B12, methylcobalamin; MTHF, methyltetrahydrofolate; P5P, pyridoxal-5-phosphate; TMG, trimethylglycine; Trp, tryptophan

#### Patient one: therapeutic program

As noted above, and following an extended discussion of the components of the therapeutic program, the patient began on some but not all of the system: (1) she eliminated all simple carbohydrates, leading to a weight loss of 20 pounds; (2) she eliminated gluten and processed food from her diet, and increased vegetables, fruits, and non-farmed fish; (3) in order to reduce stress, she began yoga, and ultimately became a yoga instructor; (4) as a second measure to reduce the stress of her job, she began to meditate for 20 minutes twice per day; (5) she took melatonin 0.5mg po qhs; (6) she increased her sleep from 4-5 hours per night to 7-8 hours per night; (7) she took methylcobalamin 1mg each day; (8) she took vitamin D3 2000IU each day; (9) she took fish oil 2000mg each day; (10) she took CoQ_10_ 200mg each day; (11) she optimized her oral hygiene using an electric flosser and electric toothbrush; (12) following discussion with her primary care provider, she reinstated HRT (hormone replacement therapy) that had been discontinued following the WHI report in 2002; (13) she fasted for a minimum of 12 hours between dinner and breakfast, and for a minimum of three hours between dinner and bedtime; (14) she exercised for a minimum of 30 minutes, 4-6 days per week.

#### Patient two: history

A 69-year-old entrepreneur and professional man presented with 11 years of slowly progressive memory loss, which had accelerated over the past one or two years. In 2002, at the age of 58, he had been unable to recall the combination of the lock on his locker, and he felt that this was out of the ordinary for him. In 2003, he had FDG-PET (fluoro-deoxyglucose positron emission tomography), which was read as showing a pattern typical for early Alzheimer's disease, with reduced glucose utilization in the parietotemporal cortices bilaterally and left > right temporal lobes, but preserved utilization in the frontal lobes, occipital cortices, and basal ganglia. In 2003, 2007, and 2013, he had quantitative neuropsychological testing, which showed a reduction in CVLT (California Verbal Learning Test) from 84%ile to 1%ile, a Stroop color test at 16%ile, and auditory delayed memory at 13%ile. In 2013, he was found to be heterozygous for ApoE4 (3/4). He noted that he had progressive difficulty recognizing the faces at work (prosopagnosia), and had to have his assistants prompt him with the daily schedule. He also recalled an event during which he was several chapters into a book before he finally realized that it was a book he had read previously. In addition, he lost an ability he had had for most of his life: the ability to add columns of numbers rapidly in his head.

He had a homocysteine of 18 μmol/l, CRP <0.5mg/l, 25-OH cholecalciferol 28ng/ml, hemoglobin A1c 5.4%, serum zinc 78mcg/dl, serum copper 120mcg/dl, ceru-loplasmin 25mg/dl, pregnenolone 6ng/dl, testosterone 610ng/dl, albumin:globulin ratio of 1.3, cholesterol 165mg/dl (on Lipitor), HDL 92, LDL 64, triglyceride 47, AM cortisol 14mcg/dl, free T3 3.02pg/ml, free T4 1.27ng/l, TSH 0.58mIU/l, and BMI 24.9.

He began on the therapeutic program, and after six months, his wife, co-workers, and he all noted improvement. He lost 10 pounds. He was able to recognize faces at work unlike before, was able to remember his daily schedule, and was able to function at work without difficulty. He was also noted to be quicker with his responses. His life-long ability to add columns of numbers rapidly in his head, which he had lost during his progressive cognitive decline, returned. His wife pointed out that, although he had clearly shown improvement, the more striking effect was that he had been accelerating in his decline over the prior year or two, and this had been completely halted.

#### Patient two: therapeutic program

The patient began on the following parts of the overall therapeutic system: (1) he fasted for a minimum of three hours between dinner and bedtime, and for a minimum of 12 hours between dinner and breakfast; (2) he eliminated simple carbohydrates and processed foods from his diet; (3) he increased consumption of vegetables and fruits, and limited consumption of fish to non-farmed, and meat to occasional grass-fed beef or organic chicken; (4) he took probiotics; (5) he took coconut oil i tsp bid; (6) he exercised strenuously, swimming 3-4 times per week, cycling twice per week, and running once per week; (7) he took melatonin 0.5mg po qhs, and tried to sleep as close to 8 hours per night as his schedule would allow; (8) he took herbs *Bacopa monniera* 250mg, *Ashwagandha* 500mg, and turmeric 400mg each day; (9) he took methylcobalamin 1mg, methyltetrahydrofolate 0.8mg, and pyridoxine-5-phosphate 50mg each day; (10) he took citicoline 500mg po bid; (11) he took vitamin C 1g per day, vitamin D3 5000IU per day, vitamin E 400IU per day, CoQ_10_ 200mg per day, Zn picolinate 50mg per day, and α-lipoic acid 100mg per day; (12) he took DHA (docosahexaenoic acid) 320mg and EPA (eicosapentaenoic acid) 180mg per day.

#### Patient three: history

A 55-year-old attorney suffered progressively severe memory loss for four years. She accidentally left the stove on when she left her home on multiple occasions, and then returned, horrified to see that she had left it on once again. She would forget meetings, and agree to multiple meetings at the same time. Because of an inability to remember anything after a delay, she would record conversations, and she carried an iPad on which she took copious notes (but then forgot the password to unlock her iPad). She had been trying to learn Spanish as part of her job, but was unable to remember virtually anything new. She was unable to perform her job, and she sat her children down to explain to them that they could no longer take advantage of her poor memory, that instead they must understand that her memory loss was a serious problem. Her children noted that she frequently became lost in mid-sentence, that she was slow with responses, and that she frequently asked if they had followed up on something she thought she had asked them to do, when in fact she had never asked them to do the tasks to which she referred.

Her homocysteine was 9.8μmol/l, CRP 0.16mg/l, 25-OH cholecalciferol 46ng/ml, hemoglobin A1c 5.3%, pregnenolone 84ng/dl, DHEA 169ng/dl, estradiol 275pg/ml, progesterone 0.4ng/ml, insulin 2.7μIU/ml, AM cortisol 16.3mcg/dl, free T3 3.02pg/ml, free T4 1.32ng/l, and TSH 2.04mIU/l.

After five months on the therapeutic program, she noted that she no longer needed her iPad for notes, and no longer needed to record conversations. She was able to work once again, was able to learn Spanish, and began to learn a new legal specialty. Her children noted that she no longer became lost in mid-sentence, no longer thought she had asked them to do something that she had not asked, and answered their questions with normal rapidity and memory.

#### Patient three: therapeutic program

She began on the following parts of the therapeutic system: (1) she fasted for a minimum of three hours between dinner and bedtime, and for a minimum of 12 hours between dinner and breakfast; (2) she eliminated simple carbohydrates and processed foods from her diet; (3) she increased consumption of vegetables and fruits, limited consumption of fish to non-farmed, and did not eat meat; (4) she exercised 4-5 times per week; (5) she took melatonin 0.5mg po qhs, and tried to sleep as close to 8 hours per night as her schedule would allow; (6) she tried to reduce stress in her life with meditation and relaxation; (7) she took methylcobalamin 1mg 4x/wk and pyridoxine-5-phosphate 20mg each day; (8) she took citicoline 200mg each day; (9) she took vitamin D3 2000IU per day and CoQ_10_ 200mg per day; (10) she took DHA 700mg and EPA 500mg bid; (11) her primary care provider prescribed bioidentical estradiol with estriol (BIEST), and progesterone; (12) her primary care provider worked with her to reduce her bupropion from 150mg per day to 150mg 3x/wk.

All 10 patients are summarized in Table 2.

**Table 2:** Summary of patients treated with the therapeutic system described

Patient	History, evaluation	Diagnosis	Status
67F 3/3	2yr memory ⇓; FH+	aMCI	Normal x 2.5 yrs; working
69M 4/3	12yr memory ⇓; FDG-PET+, NPsych+	Early AD	“Clearly improved;” working
70M 4/3	4yr memory ⇓; NPsych+, failed MemTrax	AD	Improved; MemTrax passed
75M 3/3	1yr memory ⇓	SCI	Improved; working
75F C677T	1yr memory ⇓	aMCI/early AD	Improved
55F 3/3	4yr memory ⇓	aMCI/early AD	Normal; working
72M 3/3	7yr memory ⇓	aMCI	Improved; working
55M 4/3	2yr memory ⇓	SCI	Normal; working
63F 4/3	FH dementia, mild memory ⇓	SCI	Normal, negative amyloid PET; working
60F 4/3	4yr rapid decline; MoCA 6, amyloid PET+	Late AD	Decline

F, female; M, male; 3/3, ApoE 3/3; 4/3, ApoE 4/3; C677T, the C677T mutation in methylene tetrahydrofolate reductase (MTHFR); FH, family history; aMCI, amnestic mild cognitive impairment; SCI, subjective cognitive impairment; FDG-PET+, fluorodeoxyglucose positron emission tomography interpreted as typical of Alzheimer's disease; amyloid PET+, amyloid PET scan read as abnormal, indicative of amyloid accumulation; NPsych+, quantitative neuropsychology tests showing abnormalities typical of AD; MoCA, Montreal Cognitive Assessment; MemTrax, an iPhone application that quantitates memory.

## DISCUSSION

Results from the 10 patients reported here suggest that memory loss in patients with subjective cognitive impairment, mild cognitive impairment, and at least the early phase of Alzheimer's disease, may be reversed, and improvement sustained, with the therapeutic program described here. This is the first such demonstration. However, at the current time the results are anecdotal, and therefore a more extensive, controlled clinical trial is warranted.

The results reported here are compatible with the notion that metabolic status represents a crucial, and readily manipulable, determinant of plasticity, and in particular of the abnormal balance of plasticity exhibited in SCI, MCI, and early AD. Furthermore, whereas the normalization of a single metabolic parameter, such as vitamin D3, may exert only a modest effect on pathogenesis, the optimization of a comprehensive set of parameters, which together form a functional network, may have a much more significant effect on pathogenesis and thus on function.

The therapeutic system described in this report derives from basic studies of the role of APP signaling and proteolysis in plasticity, and the imbalance in this receptor proteolysis that reproducibly occurs in Alzheimer's disease. There are numerous physiological parameters that feed into this balance, such as hormones [28, 29], trophic factors [18], glucose metabolism [30], inflammatory mediators [31], ApoE genetic status [32] sleep-related factors [33], exercise-related factors [34], and many others; therefore, the therapeutic system is designed to reverse the self-reinforcing (i.e., prionic) signaling imbalance that we have hypothesized to mediate Alzheimer's disease pathophysiology [8].

One potentially critical result from the study is the impact of the therapeutic program on the ability of the various patients to work effectively. Six of the 10 patients had had to discontinue working or were struggling with their jobs at the time of presentation, and all were able to return to work or continue working with improved performance. One additional patient had not had difficulty at work at the time of presentation, and has continued to work without difficulty. The other three patients had not worked for years, and did not begin again after treatment. The improvement in function that is required to work effectively after struggling due to cognitive decline is an important outcome of any successful therapeutic system, and is ultimately more critical to the patients than biomarker effects or test performance.

It is recognized that the system described here is an initial system, one that is likely to benefit from optimization. The system is designed to address multiple key pathogenetic mechanisms, but most of the key pathogenetic mechanisms are suboptimally affected by this initial system. This highlights multiple potential therapeutic targets, and optimizing the therapeutics for each of these targets is the goal of ongoing research and development.

It is noteworthy that the major side effect of this therapeutic system is improved health and optimal BMI (body mass index), a result in stark contrast to monopharmaceutical treatments. However, the program is not easy to follow, and none of the patients followed the entire protocol. The significant diet and lifestyle changes, and multiple pills required each day, were the two most common complaints of the patients. However, these complaints were mitigated by the fact that all of the patients had previously been made aware, either through their physicians or the media, that their prognosis was poor and their cognitive decline essentially untreatable.

One potentially important application of the therapeutic program described herein is that such a therapeutic system may be useful as a platform on which drugs that would fail as monotherapeutics may succeed as key components of a therapeutic system. Combination therapeutics have proven successful in multiple chronic illnesses, such as HIV and cancer [5].

The positive results reported here are perhaps not surprising given that therapeutic programs have proven more effective than monotherapeutics in multiple chronic illnesses, such as atherosclerotic cardiovascular disease, HIV, and cancer [5, 35]. Indeed, chronic illnesses may be more amenable to therapeutic systems than to monotherapeutics. However, the current, anecdotal results require a larger trial, not only to confirm or refute the results reported here, but also to address key questions raised, such as the degree of improvement that can be achieved routinely, how late in the course of cognitive decline reversal can be effected, whether such an approach may be effective in patients with familial Alzheimer's disease, and how long improvement can be sustained.

## In summary

- A novel, comprehensive, and personalized therapeutic system is described that is based on the underlying pathogenesis of Alzheimer's disease. The basic tenets for the development of this system are also described.
- Of the first 10 patients who utilized this program, including patients with memory loss associated with Alzheimer's disease (AD), amnestic mild cognitive impairment (aMCI), or subjective cognitive impairment (SCI), nine showed subjective or objective improvement.
- One potentially important outcome is that all six of the patients whose cognitive decline had a major impact on job performance were able to return to work or continue working without difficulty.
- These anecdotal results suggest the need for a controlled clinical trial of the therapeutic program.
